# *CALR* type 1 mutations are associated with an increased incidence of myelofibrosis in young male patients

**DOI:** 10.1007/s11845-022-03047-1

**Published:** 2022-06-07

**Authors:** Philip Weir, Andrew Hindley, Mark Catherwood, Mary Frances McMullin

**Affiliations:** 1 Department of Haematology, Belfast City Hospital, Belfast, UK; 2 Patrick G Johnston Centre for Cancer Research, Queen’s University Belfast, Belfast, UK; 3 Centre for Medical Education, Queen’s University Belfast, Belfast, UK

**Keywords:** CALR, Essential thrombocythaemia, MPN, Myelofibrosis, Myeloproliferative neoplasm

## Abstract

**Background:**

Calreticulin (*CALR*) mutations are commonly identified in patients with essential thrombocythaemia or myelofibrosis. *CALR* type 1 mutations are known to have a higher overall incidence in males but little is known about the risks of mutation subtypes on myelofibrotic change across patient age and sex.

**Aims:**

To identify differences in the incidence of myelofibrotic change within subgroups of patients with *CALR* type 1 mutations.

**Methods:**

All patients with a positive *CALR* exon 9 mutation identified within our unit between February 2016 and September 2020 were reviewed with note taken of patient sex, age at diagnosis, initial MPN diagnosis, and subsequent disease transformation.

**Results:**

In our cohort, young male patients with *CALR* type 1 mutations were shown to be at significantly increased risk of myelofibrosis compared to age matched female patients.

**Conclusions:**

Male patients have a worse myeloproliferative neoplasm phenotype than female patients with it occurring at a younger age and being more myelofibrotic in nature. Further investigation is needed into the reasons for this variability.

## Introduction

Calreticulin (*CALR*) exon 9 mutations are the second most common mutations in patients with essential thrombocythaemia (ET) and primary myelofibrosis (PMF). The overall risk of myelofibrotic change, either PMF or secondary myelofibrosis (SMF), appears to be more frequent in patients with *CALR* type 1 than type 2 mutations [1] and male patients are more likely than females to have *CALR* type 1 mutations [2].

It had been noted locally that a significant number of young male patients with *CALR* mutations appeared to have a more aggressive/myelofibrotic disease phenotype. The aim of this study was to identify if there were further differences in the incidence of myelofibrotic change within subgroups of patients with *CALR* mutations, specifically looking to see if males with *CALR* type 1 mutations were more likely to have myelofibrosis than females and if they developed it at a younger age.

## Methods

The study reviewed all patients with a positive *CALR* exon 9 mutation identified within our unit between February 2016 and September 2020. *CALR* mutations were identified as previously described [3]. Mutation sub-types were identified and compared with patient sex, age at diagnosis, initial MPN diagnosis, and subsequent disease transformation. Statistical analysis was performed with *p*-values for the total male and female patients with myelofibrosis within each age group calculated using Fisher’s exact test.

## Results

*CALR* type 1 mutations (including type 1-like mutations throughout) or type 2 mutations were identified in 123 patients and were overall more frequent in males (74 male, 49 female, *p* = 0.024) as shown in Table 1. Seventy-five (61.0%) of these patients had *CALR* type 1 mutations and 48 (39.0%) type 2. The incidence of *CALR* type 2 mutations in male compared with female patients were significantly higher in those under the age of 60 but not different in those over 60; however, further detailed analysis of myelofibrotic variances in *CALR* type 2 mutations was prevented by low patient numbers within each subgroup.

**Table 1:** Diagnoses (initial and subsequent) by age of male and female patients with *CALR* mutations

			Male (M)					Female (F)					*p*-value, M vs F				
	MPN subtypes	Total patients	Total	Age < 40	Age < 50	Age < 60	Age 60 +	Total	Age < 40	Age < 50	Age < 60	Age 60 +	Total	Age < 40	Age < 50	Age < 60	Age 60 +
*CALR* type 1	All	75	44	5	13	25	19	31	2	5	18	13	0.1333	0.2568	0.0593	0.2858	0.2888
	Initially ET	61	35	3	8	18	17	26	2	5	16	10	-	-	-	-	-
	PMF	14	9	2	5	7	2	5	0	0	2	3	-	-	-	-	-
	SMF	14	12	3	4	6	6	2	0	0	2	0	-	-	-	-	-
	Total MF	28	21	5	9	13	8	7	0	0	4	3	**0.0315**	**0.0476**	**0.0294**	0.0637	0.4501
*CALR* type 2	All	48	30	5	9	18	12	18	2	4	7	11	0.0833	0.2568	0.1655	**0.0278**	0.8348
	Initially ET	46	29	4	8	17	11	17	2	3	6	11	-	-	-	-	-
	PMF	2	1	0	0	0	1	1	0	1	0	0	-	-	-	-	-
	SMF	5	3	1	1	2	1	1	0	0	0	1	-	-	-	-	-
	Total MF	7	4	1	1	2	2	2	0	1	1	1	1.0000	1.0000	1.0000	1.0000	1.0000

Bold font indicates *p*-values of significance

*CALR type 1* calreticulin type 1 (including type 1-like mutations), *CALR type 2* calreticulin type 2, *MPN* myeloproliferative neoplasm, *Initially ET* first diagnosed with essential thrombocythaemia, *PMF* initially diagnosed with primary myelofibrosis, *SMF* subsequently diagnosed with secondary myelofibrosis, *Total MF* the total number of patients with myelofibrosis including both PMF and SMF

Of the 44 male patients with *CALR* type 1 mutations, 9 were diagnosed with PMF and 35 with ET. Twelve of those with ET subsequently transformed to SMF. Thirty-one female patients had *CALR* type 1 mutations with 5 of these being diagnosed with PMF and 26 with ET. Two transformed to SMF. The overall risk of myelofibrosis in patients with *CALR* type 1 mutations was therefore significantly higher in male than in female patients with 21 of 44 (47.7%) versus 7 of 31 (22.6%), respectively, *p* = 0.0315.

Whilst the overall risk of myelofibrosis with *CALR* type 1 mutations is highest in males, this risk appears to vary between age groups. The mean age of developing myelofibrosis, either PMF or SMF, in the cohort was 61 overall with it being 59 for males and 66 for females. The median was 62 overall with a median age of 60 for males and 65 for females. These findings indicate that myelofibrosis occurs at a younger age in male patients who have *CALR* type 1 mutations.

To consider this further, the data was split by age group and is summarised in Table 1. Of the 32 patients aged 60 or over with *CALR* type 1 mutations, 19 were male and 13 were female. Eight of the male and 3 of the female patients had myelofibrosis. Whilst in keeping with the known increased frequency of males having *CALR* type 1 mutations, the risk of myelofibrosis between sexes was not statistically different in this older age group, *p* = 0.4501.

The risk of myelofibrosis was also not quite statistically different when looking at those with *CALR* type 1 mutations under 60 years of age overall at 0.0637; however, myelofibrosis became increasingly common in the male sex among younger age groups. Of the 18 patients under 50 years old, 13 were male and 5 were female. All females had ET without progression but 9 of the males had developed myelofibrosis, *p* = 0.0294. Of the 7 patients under 40 years old, the 2 females had ET but all 5 males had myelofibrosis, *p* = 0.0476.

## Discussion

This study highlights that in our cohort, *CALR* type 1 mutations are associated with an overall increased incidence of myelofibrosis in male compared with female patients. This variation is particularly evident in those diagnosed at a young age where despite low patient numbers, there is a statistically increased risk of myelofibrosis among male patients under the age of 40 which persists in those under 50 but becomes less relevant in those diagnosed later in life.

In our cohort, all 9 patients with myelofibrosis under the age of 50 were male and this finding does not appear to be an outlier. Although not specifically investigated in their study, previously published data by Cabagnols et al. [4] showed a similar trend in their cohort, with males accounting for 83.3% of patients under 50 with both *CALR* type 1 mutations and myelofibrosis. This was despite them making up only 53.4% of *CALR* type 1 mutation positive patients overall.

The reason for these variations in incidence and MPN phenotype in younger age groups is not currently understood but may relate to hormonal variations. The incidence of myelofibrosis between male and female patients being significantly different in those under 50 years old but not significant beyond this age appears to correlate with the age of menopause.

High oestrogen levels present in premenopausal females may be protective against both *CALR* type 1 mutation or allele burden, and transformation to myelofibrosis. Tamoxifen, a selective oestrogen receptor modulator, has long been known to exhibit a complex spectrum of both oestrogen agonistic and antagonistic effects depending on the specific tissue affected [5]. The recent Tamarin study showed that tamoxifen could induce apoptosis of *CALR* mutated haematopoietic progenitors, and based on preclinical studies, this appeared to work by modulation of oestrogen receptor alpha [6]. It remains unclear if Tamoxifen’s modulatory role in this area is oestrogen agonistic or antagonistic, but these results give further weight to oestrogen’s relevance in MPN associated with *CALR* mutations.

## Conclusion

Whilst it is already known that *CALR* type 1 mutations are more common in males, this study is the first to demonstrate that even within the demographic of those with *CALR* type 1 mutations, male patients have a worse MPN phenotype than female patients with it occurring at a younger age and being more myelofibrotic in nature. The potential reasons for this variability as discussed could lead to new opportunities for treatment and warrant further investigation.
